# Associations Between Commercial App Use and Physical Activity: Cross-Sectional Study

**DOI:** 10.2196/17152

**Published:** 2020-06-03

**Authors:** Jasmine Maria Petersen, Eva Kemps, Lucy K Lewis, Ivanka Prichard

**Affiliations:** 1 SHAPE Research Centre Flinders University Adelaide Australia; 2 Caring Futures Institute College of Nursing and Health Sciences Flinders University Adelaide Australia; 3 School of Psychology, College of Education, Psychology and Social Work Flinders University Adelaide Australia

**Keywords:** physical activity, mobile applications, social networking

## Abstract

**Background:**

In today’s society, commercial physical activity apps (eg, Fitbit and Strava) are ubiquitous and hold considerable potential to increase physical activity behavior. Many commercial physical activity apps incorporate social components, in particular app-specific communities (allowing users to interact with other app users) or the capacity to connect to existing social networking platforms (eg, Facebook or Instagram). There is a growing need to gain greater insights into whether commercial physical activity apps and specific components of these apps (social components) are beneficial in facilitating physical activity.

**Objective:**

This study aimed to examine the relationship between the use of commercial physical activity apps and engagement in physical activity. The social components of commercial physical activity apps (app-specific communities and existing social networking platforms) were also explored. This involved isolating specific features (eg, sharing, providing, and receiving encouragement, comparisons, and competitions) of app-specific communities and existing social networking platforms that were most valuable in facilitating physical activity.

**Methods:**

A cross-sectional web-based survey was conducted. Participants were 1432 adults (mean age 34.1 years, 1256/1432, 88.00% female) who completed measures assessing physical activity, the use of commercial physical activity apps, and engagement with app-specific communities and existing social networking platforms.

**Results:**

Overall, 53.14% (761/1432) of the sample reported engaging with a commercial physical activity app. The most commonly used apps were Fitbit (171/761, 22.5%), Strava (130/761, 17.1%), and Garmin (102/761, 13.4%). The use of physical activity apps was significantly associated with physical activity. Notably, the use of app-specific communities and existing social networking platforms facilitated significantly greater engagement in physical activity. The features of app-specific communities that were most beneficial in promoting engagement in physical activity were providing encouragement to a partner, receiving encouragement from close friends and family, and engaging in competitions with members of public app-specific communities. In relation to existing social networking platforms, sharing physical activity posts predicted engagement in physical activity.

**Conclusions:**

The findings indicate that app-specific communities and existing social networking platforms are components of apps that are fundamental in facilitating physical activity. They further suggest that commercial physical activity apps afford high population level reach and hold great potential to promote engagement in physical activity, an important public health consideration.

## Introduction

### Background

Physical activity confers many health benefits, including a reduced risk of cardiovascular disease, hypertension, osteoporosis, diabetes mellitus, obesity, mental illness, and premature mortality [1-3]. Despite this, globally, 1.4 billion adults (28%) are not meeting physical activity guidelines (150 min of moderate to vigorous physical activity per week), a figure that continues to rise [4]. This highlights the need to develop scalable interventions to increase physical activity.

Physical activity mobile apps present an innovative approach to promote engagement in physical activity due to their widespread reach, accessibility, and convenience. Recently, there has been exponential growth in the availability of commercial physical activity apps (eg, Fitbit, Strava, and Garmin) [5]. However, much of the previous research examining physical activity apps has focused on apps developed by researchers as opposed to commercially available apps [6,7]. This presents a shortcoming of research to date, such that despite the accessibility and ubiquity of commercial apps, there is limited literature exploring their use and influence on physical activity. This indicates the need to gain greater insight into the use of commercial apps to ascertain their capacity to increase levels of physical activity and, thus, improve public health.

A growing body of research examining the content of commercial apps has identified that social features are an increasingly ubiquitous component [6,8-10]. That is, many commercial apps incorporate app-specific communities, allowing users to interact with other app users by sharing physical activity data, receiving or providing encouragement (eg, likes and comments), and engaging in competitions or comparisons [8]. However, to date, there has been little examination of app-specific communities, and, in particular, the association between engagement with the features of these communities (eg, sharing and competitions) and physical activity. Insights into app-specific communities is important to ascertain their value in promoting engagement in physical activity and crucial for the development of future physical activity interventions.

Content analyses of commercial apps have identified that many physical activity apps also have the capacity to connect to existing social networking platforms such as Facebook or Instagram [6,8-10]. This has been suggested to be an important component of an app, given that a recent review [7] identified that the use of existing social networking platforms in conjunction with apps enhances engagement [11]. However, the review [7] also documented that this research area is in its infancy, and there is a need to gain greater insights into how to optimally harness existing social networking platforms in conjunction with physical activity apps. This requires identifying the features of existing social networking platforms (eg, social interaction and comparisons) that are associated with app engagement and, thus, physical activity.

### Objectives

To our knowledge, no previous study has comprehensively examined commercial physical activity apps and specifically, how the social components of these apps (app-specific communities or existing social networking platforms) may be associated with physical activity. This is important given the increasing prevalence of commercial physical activity apps together with the need to isolate components of apps that are linked to physical activity engagement. Thus, the first aim of this study was to gain a comprehensive understanding of the use of commercial physical activity apps and their relationship with physical activity. The second aim was to explore the value of app-specific communities and existing social networking platforms in facilitating physical activity. More specifically, we sought to ascertain the features of app-specific communities and existing social networking platforms that were used and how these were associated with frequency of app use and engagement in physical activity.

## Methods

### Study Design and Participants

A web-based cross-sectional survey was conducted. Participants were recruited via the Discipline of Psychology’s web-based research participation system, paid Facebook advertising, and free advertisements placed on social networking platforms (eg, Facebook, Instagram, and Twitter) for a study on *Physical Activity and Online Social Networking*. Ethical approval was obtained from the University Social and Behavioral Research Ethics Committee (protocol no. 8232). All participants provided informed consent electronically. Participants were adults, ≥18 years, and proficient in English.

### Procedure

Participants completed a web-based survey through the Qualtrics platform between February and April 2019. The survey took approximately 30 min to complete and incorporated the measures listed below in the order of presentation. As a token of appreciation, participants could enter a raffle to win 1 of 5 AUD $25 (USD $15) shopping gift vouchers.

### Measures

#### Demographics

Participants were invited to report their age, gender identity, and ethnicity.

#### Regular Structured Physical Activity

Regular structured physical activity was assessed following the methods of Prichard and Tiggemann [12]. Participants were invited to self-report the type, duration, and frequency of structured physical activity or sports they generally engaged in on a weekly basis. The total number of minutes of physical activity engaged in per week was then calculated by multiplying each activity’s frequency by its duration. Separate physical activity totals were calculated according to the type of physical activity listed, specifically individual physical activities (eg, walking or running), gym-based activities (eg, gym classes), or sports-based activities (eg, netball or football).

#### Current Physical Activity App Use

Participants were asked to self-report their current use of physical activity apps, defined as apps that have the capacity to track or monitor physical activity (eg, steps or distance) or provide guided training or workouts. In particular, participants were asked to self-report using an open-ended response format, the name of the physical activity app they were currently using most frequently (main physical activity app, eg, Strava), the physical activity or sport they were using the app for, and their level of engagement with the app (number of times used per week). The apps were categorized according to their capabilities, including tracking, providing guided workouts, tracking plus providing guided workouts, or other (eg, scheduling gym classes or immersive games). The types of physical activity the apps were used for were classified as all daily activities, individual activities (eg, running, cycling, or walking), group-based activities (eg, netball, soccer, or football), gym-based activities (eg, fitness classes or personal training), or a combination of different activities (individual, group-based and gym-based activities).

#### Engagement With an App-Specific Community

In relation to the main physical activity app participants were currently using, they were asked to self-report their engagement with the features of the app-specific community. This included specifying how frequently on a 6-point Likert scale ranging from 0 (*never*) to 5 (*very often*) they engaged with specific features of the app community, such as sharing physical activity posts, liking and/or providing positive comments on others’ posts, receiving likes and/or positive comments, comparing their physical activity performance with others, and engaging in competitions. Furthermore, participants were asked to indicate the frequency that they engaged with the aforementioned features with specific members of their app community, including partners, family, close friends, peers, public app-specific community members, and work colleagues. Example items include *Within the main physical activity app you are currently using, how often do you share posts relating to your physical activity performance with a partner?* and *Within the main physical activity app you are currently using, how often do you Like/Kudos/Cheer and/or provide positive comments on physical activity posts from close friends?* As the networks within each feature were highly correlated (α=.74), a composite score was calculated for the use of each specific feature of the app community (eg, sharing) across the different networks (eg, peers and family) while also examining the independent influence of engaging with specific networks in relation to each feature.

Participants who specified that the main physical activity app they were using incorporated an app-specific community but reported that they did not engage with it were provided with an open-ended question to determine the underlying rationale for this. Preliminary themes were established by the first author, and the responses were subsequently categorized by 2 independent coders.

#### Engagement With Existing Social Networking Platforms in Relation to Physical Activity

Participants were also asked to self-report their physical activity-related use of existing social networking platforms on measures developed for this study. Specifically, participants were asked to specify on a 6-point Likert scale how frequently (from *never*=0 to *very often*=5) they share physical activity posts, like and/or provide positive comments on others’ posts, receive likes and/or positive comments, and compare their physical activity performance with others’ physical activity posts on Facebook, Instagram, and Twitter (plus the option to specify other platform(s)). Example items included *How often do you share physical activity posts on the following social networking platforms?* and *How often do you like and/or provide positive comments on physical activity posts from other people on the following social networking platforms?* A composite score was calculated for the use of each specific feature (eg, sharing) across the different social networking platforms (eg, Facebook and Instagram).

### Statistical Analysis

Data were analyzed using Statistical Package for the Social Sciences version 25 (IBM, Corp). Significance for all analyses was set at *P*<.05 (2-tailed). Overall, the study variables (with the exception of app engagement) did not deviate substantially from normality based on skewness, kurtosis, or histogram examination. Therefore, parametric tests were used for all analyses, except those that included the variable app engagement for which a nonparametric test (Kruskal-Wallis test) was used.

Descriptive statistics were used to generate demographic information. A series of independent samples *t* tests and chi-square analyses were conducted to determine differences between app users and nonusers in age, gender identity, ethnicity, and minutes of physical activity per week. Chi-square analyses were used to identify differences in app use (ie, the most commonly used apps, the capabilities of the apps used, and the activity app is used for) based on demographics (age and gender identity). A Kruskal-Wallis test with pairwise comparisons using the Dunn-Bonferroni correction was conducted to examine the relationship between app engagement (frequency of app use per week) and physical activity.

Kruskal-Wallis tests were also conducted to examine the relationships between the use of specific features of app-specific communities and app engagement among app users. In addition, 1-way analyses of variance were performed to determine differences in engagement with features of app-specific communities based on age, capabilities of the app used, and activity the app was used for, but not for gender identity (because of the small proportion of men). The aforementioned analyses were repeated using specific features of existing social networking platforms. Independent samples *t* tests and chi-square analyses were also used to determine differences between users and nonusers of the app-specific communities and existing social networking platforms.

Finally, a multiple linear regression was conducted to explore the predictors of physical activity among app users. The regression model incorporated the frequency of app usage and all features of both app-specific communities (including specific networks) and existing social networking platforms. Demographic characteristics (age, gender identity, and ethnicity) were incorporated as control variables.

## Results

### Sample

In total, 1640 individuals began the survey, 208 of whom did not complete it (a response rate of 87.3%), resulting in a final sample of 1432 participants. The sample had a mean age of 34.1 years (range 18-83 years) and comprised predominately female participants (1256/1432, 88.00%). Overall, the sample engaged in high levels of structured physical activity (mean 266.8 min per week, SD 219.8), and 53.14% (761/1432) reported currently engaging with a physical activity app. Table 1 presents the demographic characteristics of app users and nonusers. There were no significant differences between the 2 groups (app users and nonusers) in relation to age, gender identity, or ethnicity. However, app users engaged in significantly more structured physical activity per week than nonusers (Table 1). Overall, among those who reported engaging in physical activity, participants predominately engaged in individual physical activities (eg, walking or running, 619/858, 72.2%), followed by gym-based activities (eg, gym classes, 324/620, 52.2%), and sports-based activities (eg, netball or football, 88/324, 27.2%). This did not differ based on whether participants used an app. Relatedly, participants spent the most time engaging in individual activities per week (mean 133.6 min, SD 176.5), followed by sports-based activities (mean 113.5 min, SD 110.7), and gym-based activities (mean 83.2 min, SD 94.9). Again, this was consistent across app users and nonusers (Table 1).

**Table 1:** Sample characteristics of physical activity app users and nonusers (n=1432).

Characteristic		App users (n=761)		Nonusers (n=671)		*P* value^a^		Effect size, *Φ*		Effect size, Cohen *d*	
**Age (years), n** **(%)**						.42		0.12		N/A^b^	
	18-25		243 (32.0)		257 (38.3)						
	>25-30		115 (15.0)		76 (11.4)						
	>30-40		190 (25.0)		119 (17.7)						
	>40		208 (27.3)		214 (31.9)						
**Gender identity, n** **(%)**						.94		0.006		N/A	
	Female		668 (88.0)		588 (88.0)						
	Male		84 (11.0)		73 (11.0)						
**Ethnicity, n** **(%)**						.67		0.05		N/A	
	White		682 (89.6)		581 (86.6)						
	Asian		35 (4.6)		37 (5.5)						
	Indian		10 (1.3)		13 (1.9)						
	Other		34 (4.5)		40 (6.0)						
**Structured physical activity (min per week), mean (SD)**											
	Overall structured physical activity		309.0 (214)		219.0 (216)		<.001		N/A		0.42
	Individual activities (n=858)		141.9 (179.4)		120.6 (171.3)		.21		N/A		0.08
	Sport-based activities (n=324)		115.5 (120.4)		110.7 (96.4)		.61		N/A		0.06
	Gym-based activities (n=620)		77.4 (79.8)		92.8 (115.3)		.23		N/A		0.09

^a^Statistical significance is represented by *P*<.05.

^b^N/A: not applicable.

### Physical Activity App Use

Multimedia Appendix 1 presents the physical activity apps that were most commonly used. Fitbit (171/761, 22.5%), followed by Strava (130/761, 17.1%) and Garmin (102/761, 13.4%) were the most popular apps, and this did not differ by age or gender identity. Participants most commonly engaged with apps that had the capacity to exclusively track behaviors and predominately used apps for individual activities (eg, running or walking). This was consistent across age and gender identities.

The greatest proportion of participants reported using their physical activity app on 7 occasions per week (296/761, 39.0%), followed by use on 3 occasions (102/761, 13.4%) and more than 7 occasions (70/761, 9.2%) per week. A Kruskal-Wallis Test comparing weekly physical activity duration revealed a statistically significant difference (*P*=.006) across levels of app usage. Specifically, pairwise comparisons identified that participants who used the app on 6 occasions per week engaged in significantly higher levels of structured physical activity (median 491.9 min) than those who used the app on 2 occasions per week (median 297.4 min; *P*=.003). Overall, participants who used an app 6 times per week engaged in the highest levels of structured physical activity.

### Use of Social Components of Physical Activity Apps

Among app users, 3.4% (26/761) used app-specific communities exclusively, 59.9% (456/761) used existing social networking platforms exclusively, and 22.0% (167/761) used both app-specific communities and existing social networking platforms. This did not differ significantly by age, gender identity, ethnicity, capabilities of the app used (eg, tracking), the type of physical activity the app was used for, or frequency of app usage per week.

### Physical Activity App Use in Conjunction With an App-Specific Community

Among app users, 59.0% (447/761) reported that the physical activity app they were currently using incorporated an app-specific community. Of these, 43.1% (193/447) reported engaging with the community. Participants who reported engaging with the app-specific communities predominantly used Strava (80/193, 41.5%), Fitbit (40/193, 20.7%), and Garmin (13/193, 6.7%). Table 2 shows that the distribution of age was significantly different between app community users and nonusers. Specifically, app community users were predominately >30 years. App community users also engaged in significantly more structured physical activity per week than nonusers (t_445_=2.62; *P*=.009; *d*=0.25). However, there were no significant differences between users and nonusers of the app-specific community in relation to gender identity, ethnicity, capabilities of the app used (eg, tracking), the type of physical activity the app was used for, or app usage per week. Among participants who reported not engaging with the app-specific community (254/447, 57.0%), the reasons identified were privacy or security concerns, negative attitudes toward the use of the community, considered unnecessary, lack of support, beliefs regarding the nature of physical activity, disinterest in others’ physical activity performance, and use of an alternative social network. Of these, the most commonly cited reasons were that the use of the community was considered unnecessary (83/249, 33.4%), disinterest in others’ physical activity performance (50/246, 20.3%), and privacy or security concerns (43/246, 17.5%).

Among participants who engaged with the app-specific community (n=193), users most frequently used features that allowed the sharing of physical activity performance, providing encouragement to others’ physical activity posts (eg, likes or positive comments), and receiving encouragement on one’s own posts. These features were most frequently reported to be used with networks that were close friends or peers. There were no significant differences in engagement with features of app-specific communities across age, levels of app usage or according to capabilities of the app used, or the activity the app was used for.

**Table 2:** Sample characteristics of app-specific community users and nonusers (n=447).

Characteristic		Community users (n=193)	Nonusers (n=254)	*P* value^a^	Effect size, *Φ*	Effect size, *Cohen d*
**Age (years),** **n** **(%)**				.005	0.16	N/A^b^
	18-25	34 (17.7)	77 (30.4)			
	>25-30	29 (15.1)	46 (18.2)			
	>30-40	59 (30.6)	65 (25.7)			
	>40	70 (36.2)	65 (25.7)			
**Gender identity, n (%)**				.05	0.09	N/A
	Female	159 (82.4)	229 (90.2)			
	Male	29 (15.0)	24 (9.4)			
**Ethnicity, n (%)**				.45	0.11	N/A
	White	177 (91.7)	231 (90.9)			
	Asian	9 (4.7)	9 (3.5)			
	Indian	2 (1.0)	4 (1.6)			
	Other	5 (2.6)	10 (4.0)			
Structured physical activity (min per week), mean (SD)		357.6 (217.7)	305.4 (196.3)	.009	N/A	0.25
**Type of app, n** **(%)**				.26	0.09	N/A
	Tracking	184 (95.3)	234 (92.1)			
	Guided workouts	5 (2.6)	14 (5.5)			
	Tracking and workouts	3 (1.6)	2 (0.8)			
	Other (booking classes or immersive games)	1 (0.5)	4 (1.6)			
**Physical activity app is used for, n** **(%)**				.23	0.11	N/A
	All daily activity	18 (9.3)	39 (15.3)			
	Individual activities	154 (80.0)	184 (72.4)			
	Group-based activities	0 (0.0)	1 (0.4)			
	Gym-based activities	10 (5.2)	18 (7.1)			
	Combination of individual and group-based and gym-based activities	6 (3.1)	6 (2.4)			

^a^Statistical significance is represented by *P*<.05.

^b^N/A: not applicable.

### Physical Activity App Use in Conjunction With Existing Social Networking Platforms

Among app users, 82.0% (624/761) reported using existing social networking platforms (Facebook, Instagram, or Twitter) in relation to physical activity. There were no significant differences between users and nonusers of existing social networking platforms in relation to age, gender identity, or ethnicity. Participants who used existing social networking platforms engaged in significantly more structured physical activity than those who did not (t_672_=2.9; *P*=.004; *d*=0.44). The features of existing social networking platforms that were most frequently used were providing encouragement on others’ physical activity posts, followed by receiving encouragement on one’s own physical activity posts (eg, likes or comments). Notably, there were significant differences in the frequency of engagement with features of existing social networking platforms based on age, both in terms of sharing physical activity posts (*F*_3,666_=5.37; *P*=.001) and engaging in comparisons (*F*_3,666_=19.0; *P*<.001). Specifically, participants aged 18-25 years shared posts to existing social networking platforms significantly less frequently than all other age groups. In addition, participants >40 years made significantly fewer comparisons relative to all other age groups. However, there were no significant differences in the frequency of engagement with features of existing social networking platforms across the frequency of app usage or according to the capabilities of the app used, or the activity the app was used for.

### Exploring Predictors of Physical Activity

The regression model accounted for 42.6% of the variance in structured physical activity *(R*^2^=0.426) and was significant (*F*_38,96_=1.87; *P*<.01). The following variables were significant positive predictors of structured physical activity: frequency of app use (β=.25; *P*=.009), providing encouragement to a partner (β=.52; *P*=.005), receiving encouragement from close friends (β=.59; *P*=.01) and family (β=.48; *P*=.02), engaging in competitions with members of a public app-specific community (β=.38; *P*=.001), and sharing posts to existing social networking platforms (β=.31; *P*=.004). In addition, the following variables were significant negative predictors of structured physical activity: sharing physical activity posts with a partner (β=−.40; *P*=.007), providing encouragement to close friends (β=−.57; *P*=.01), receiving encouragement from members of a public app-specific community (β=−.35; *P*=.04), and engaging in competitions with a partner (β=−.30; *P*=.04, Table 3).

**Table 3:** Multiple regression analysis examining predictors of structured physical activity among app users (n=761).

Variable			β	*t* test	*P* value^a^
Gender identity			−.05	−0.62	.53
Age			−.02	−0.24	.81
Ethnicity			-.05	-0.48	.63
App engagement (frequency)			.25	2.64	.009
**App-specific communities**					
	**Sharing posts**				
		Partner	−.40	−2.74	.007
		Family	−.18	−1.09	.28
		Close friends	.08	0.50	.62
		Peers	.02	0.14	.88
		Public app community	−.02	−0.16	.87
		Colleagues	−.15	−1.24	.21
	**Providing encouragement**				
		Partner	.52	2.90	.005
		Family	−.08	−0.45	.64
		Close friends	−.57	−2.50	.01
		Peers	.19	0.96	.34
		Public app community	.29	1.97	.05
		Colleagues	.10	0.53	.59
	**Receiving encouragement**				
		Partner	−.14	−0.84	.40
		Family	.48	2.28	.02
		Close friends	.59	2.41	.01
		Peers	.01	0.07	.94
		Public app community	−.35	−1.98	.04
		Colleagues	−.15	−0.81	.41
	**Engagement in competitions**				
		Partner	−.30	−2.0	.04
		Family	−.06	−0.40	.69
		Close friends	−.16	−1.16	.24
		Peers	−.18	−1.25	.21
		Public app community	.38	3.27	.001
		Colleagues	−.07	−0.55	.57
	**Engagement in comparisons**				
		Partner	.15	0.93	.35
		Family	−.04	−0.24	.81
		Close friends	−.17	−1.05	.29
		Peers	.15	0.93	.35
		Public app community	.01	0.06	.94
		Colleagues	.06	0.35	.72
	**Existing social networking platforms**				
		Sharing posts	.31	2.92	.004
		Providing encouragement	−.07	−0.63	.52
		Receiving encouragement	.16	1.43	.15
		Engagement in comparisons	.02	0.20	.83

^a^Statistical significance is represented by *P*<.05.

## Discussion

### Principal Findings

This study aimed to provide a comprehensive examination of the use of commercial physical activity apps and the relationship between app usage and physical activity. In addition, we sought to explore the use of social components of apps (app-specific communities and existing social networking platforms) and their value in facilitating engagement in physical activity. This study is timely, given the ubiquity of commercial physical activity apps coupled with the need to understand how specific components of these apps may be beneficial in facilitating physical activity.

Overall, the findings demonstrate that the use of physical activity apps is common, with over half of the participants reporting that they currently use a physical activity app. Our findings are consistent with Krebs and Duncan [13], who reported that in a large, diverse US sample (50% female), 58.2% had downloaded a health-related app, of which 52.8% used the app to track physical activity. This reflects both the omnipresence of commercial physical activity apps and their capacity to have high-population level reach. Notably, physical activity app users engaged in significantly more structured physical activity, consistent with findings from a previous study documenting that physical activity app users were 27% more likely to engage in physical activity than nonusers [14]. In this study, app users predominantly used Fitbit, Strava, and Garmin, with the primary function of these apps being to track or monitor behavior. This may explain the higher levels of physical activity among app users, given that self-monitoring is a behavior change technique consistently associated with increased physical activity [15,16]. These findings suggest that commercial physical activity apps may have great potential to influence physical activity. However, it must be acknowledged that the causality of the relationship between app use and physical activity is presently unclear, in that those who engage in high levels of physical activity may be attracted to apps to monitor their behavior. Longitudinal research examining app use and physical activity over time is needed to ascertain the direction of this relationship.

This study identified that participants most commonly engaged with physical activity apps on 7 occasions per week, and in line with previous research, the frequency of app usage was significantly associated with physical activity [11]. Interestingly, participants who used the apps on 6 occasions per week engaged in the highest duration (minutes) of structured physical activity per week. This indicates that relatively high app use is associated with high levels of physical activity, reflective of the previously cited dose-response relationship between app use and behavioral outcomes (eg, physical activity) [17]. The present findings further highlight that app usage is an important consideration in appropriately leveraging apps to promote engagement in physical activity. This emphasizes the importance of examining specific components of apps that may be harnessed to increase app usage, namely social components.

A novel aspect of this study was its comprehensive examination of the social components (app-specific communities and existing social networking platforms) of commercial physical activity apps. Interestingly, the use of the social components of apps differed markedly, such that most app users engaged exclusively with existing social networking platforms (in relation to physical activity, 456/761, 59.9%), whereas far fewer engaged with both app-specific communities and existing social networking platforms (167/761, 22.0%) or app-specific communities exclusively (26/761, 3.4%). This is perhaps not surprising given that existing social networking platforms are immensely popular, afford widespread reach, and achieve high levels of sustained engagement [18]. Age is another factor that may have contributed to the difference in the use of the social components of apps, such that users of app-specific communities were predominately >30 years (66.8%); by contrast, age was not associated with the use of existing social networking platforms. This suggests that app-specific communities are most appropriate for a specific subgroup of the population (>30 years), whereas existing social networking platforms could be harnessed for the population more broadly. Relatedly, future research could consider examining other factors that may influence the use of app-specific communities and/or existing social networking platforms, such as one’s presence on social networking platforms (ie, those with vs without an established social networking presence).

Despite the differences in the usage of app-specific communities and existing social networking platforms, the social features across both were used similarly, with providing and receiving encouragement the most popular features. Interestingly, the findings also indicate that demographic characteristics may be linked to the likelihood that individuals will utilize specific features. For example, in relation to existing social networking platforms, those aged 18-25 years shared posts less frequently than all other age groups, whereas those aged >40 years engaged in comparisons less frequently relative to all other age groups. This is consistent with a recent review [19] of mobile health interventions documenting that preferences for social features that facilitated comparisons, competitions, or social support varied among participants and purported that this may be linked to individual differences (eg, competitiveness). When leveraging the features of social components of physical activity apps (eg, existing social networking platforms), a 1-size-fits-all approach is not appropriate, and individual differences must be considered. Future research may usefully extend this understanding by examining whether other individual difference factors such as psychological characteristics (competitiveness and social comparisons) may influence the use of specific features.

Notably, the use of app-specific communities and existing social networking platforms was associated with significantly higher engagement in structured physical activity. This is a novel finding given that it suggests that the social components of apps may play a fundamental role in facilitating engagement in physical activity. This may be attributed to the unique capacity of app-specific communities and existing social networking platforms to generate social support [20], an important determinant of engagement in physical activity [21,22]. Another explanation is that individuals who engage in more physical activity have more content to share and are more likely to engage with the social components of apps, which, in turn, may foster supportive interactions. This study demonstrates the value of social components of apps in facilitating physical activity and the need to further examine app-specific communities and existing social networking platforms in future research.

Finally, the regression analysis revealed that the frequency of app usage was a significant predictor of structured physical activity, indicating the need to determine strategies that will facilitate app use. Interestingly, in relation to app-specific communities, providing encouragement to a partner, receiving encouragement from close friends and family, and engagement in competitions with members of public app-specific communities were positive predictors of physical activity. Conversely, sharing physical activity posts and engaging in competitions with a partner, providing encouragement to close friends, and receiving encouragement from members of public app-specific communities were negative predictors of physical activity. These findings indicate that receiving encouragement from strong ties (close friends and family) is beneficial in facilitating engagement in physical activity, whereas receiving encouragement from weak ties (public app-specific communities) is not. This is consistent with previous research showing that strong ties provide emotional support (encouragement, empathy) [23,24], which is linked to improvements in health outcomes [25], and an increased likelihood that one will initiate and maintain engagement in physical activity [26]. Conversely, weak ties often only provide informational support (advice or suggestions) [23,24], shown to be negatively associated with health behavior [27], and this has been attributed to receiving information or advice that is unwanted or in surplus [28]. The findings do, however, suggest that engaging in competitions with weak ties (public app-specific communities) is advantageous in promoting physical activity, whereas engaging in competitions or behaviors that may generate competitions (sharing physical activity posts) with strong ties (eg, partner) is negatively associated with physical activity. This is in line with existing research documenting that comparisons (generating competitions) with strong ties elicits greater pressure and fear of experiencing shame and embarrassment, ultimately decreasing the likelihood that one will engage in the behavior [29,30]. Together, these findings provide an important understanding of the specific features (and networks) of app-specific communities that are most beneficial in facilitating physical activity, and thus should be leveraged in future app-based interventions.

The regression analysis also showed that in relation to existing social networking platforms, sharing physical activity posts positively predicted engagement in physical activity. This is perhaps not surprising given that a recent study [27] found that sharing posts related to tracked health information (eg, physical activity, sleep, or calories) to existing social networking platforms is positively associated with social support, and this, in turn, predicts engagement in the associated health behavior. This highlights the need for future research to further explore the use of existing social networking platforms in relation to physical activity apps and physical activity behavior. In addition, future studies could explore how different social networking environments (eg, network size or composition) may interact with apps to influence physical activity.

### Implications

Our findings have important implications for informing the design of future app-based interventions. They demonstrate that commercial physical activity apps, in particular, those that facilitate self-monitoring (eg, Fitbit, Strava, and Garmin) hold great potential to promote engagement in physical activity. The convenience, accessibility, and affordability of commercial apps coupled with their capacity to facilitate physical activity highlight that future app-based interventions should harness commercial apps, as opposed to previous interventions that have predominately incorporated researcher-developed apps [7]. The findings also indicate that the social components of apps are important in promoting physical activity and, thus, fundamental in the development of effective app-based interventions. More specifically, in relation to app-specific communities, receiving encouragement from close friends and family, providing encouragement to a partner, and engagement in competitions with members of public app-specific communities were shown to be the most beneficial features in facilitating physical activity and, thus, should be leveraged to maximize effectiveness. However, relatively few app users engaged with app-specific communities (193/447, 43.1%), with commonly cited barriers to using the communities, including disinterest and privacy or security concerns. Nevertheless, app-specific communities show great potential in facilitating physical activity, and thus, these barriers must be considered and overcome in the design of future apps and app-based interventions. The findings also suggest that existing social networking platforms are commonly used in relation to physical activity, and notably, sharing physical activity posts to these platforms predicts engagement in physical activity. Thus, existing social networking platforms will be an important component of future app-based interventions given their capacity to achieve high levels of use and promote engagement in physical activity.

This study also has important implications for guiding future research. Experimental evidence is now needed to isolate the influence of the social components of apps and their associated features on physical activity. Future research should also endeavor to ascertain the mechanisms (eg, social support and self-efficacy) underlying the capacity of app-specific communities and existing social networking platforms to facilitate physical activity. Finally, longitudinal research is needed to determine the value of app-specific communities and existing social networking platforms in promoting sustained app use, and thus prolonged engagement in physical activity.

### Limitations

As with all studies, there are a number of limitations that need to be acknowledged. First, the sample consisted predominantly of white women, and the participants’ origin (country/region) was unknown. These 2 sample characteristics limit the generalizability of the findings to the population more broadly. Nevertheless, app usage rates were similar to those previously reported in a large, diverse US sample [13]. Second, the sample as a whole engaged in high levels of structured physical activity, indicative of self-selection bias, and, thus, may not be representative of the general population. Third, participants self-reported structured physical activity, which may have resulted in under- or overreporting. In addition, the assessment of structured physical activity did not capture incidental physical activity, which is often recorded by apps that track or monitor daily or individual physical activities. Thus, future research should consider using accelerometer-derived measures of physical activity in conjunction with self-report measures that have the capacity to capture both structured and incidental physical activity. Finally, there are a number of factors that may influence app use and/or the association between app use and physical activity such as socioeconomic status (SES), fitness device ownership, and overall engagement with the app-specific community, which were not measured in this study. Many physical activity apps are often used in conjunction with fitness devices (eg, Fitbits and Apple Watches), which may be too expensive for some individuals. As such, future research could examine potential interactions between SES, fitness device ownership, app use, and physical activity.

### Conclusions

Notwithstanding these limitations, this study provides an important contribution to existing literature by comprehensively exploring the use of commercial physical activity apps and their associated social components in a large cross-sectional sample. The findings indicated that the use of commercial physical activity apps facilitates engagement in physical activity and, therefore, have great potential to disseminate scalable interventions to improve health behavior. This study also provided a nuanced insight into app-specific communities and existing social networking platforms, identifying that they are components of apps that are valuable in promoting physical activity, and should be harnessed in the development of future app-based interventions. Together, these findings highlight the importance of further examining the social components of apps and gaining an understanding of the mechanisms underlying their influence on physical activity. In so doing, this study has demonstrated that commercial physical activity apps afford high population level reach and hold great potential to facilitate engagement in physical activity. Thus, future interventions aimed at increasing physical activity should further explore commercial physical activity apps and their associated social components.

## Abbreviations

SES: socioeconomic status

## Appendix

Multimedia Appendix 1 Physical activity app use of the sample (n=761).
