# Supplementary material for: Associations Between Commercial App Use and Physical Activity: Cross-Sectional Study
Source: J Med Internet Res. 2020 Jun 3;22(6):e17152. doi: 10.2196/17152 (PMC7301264; doi:10.2196/17152)
Supplement: Multimedia Appendix 1 [file jmir_v22i6e17152_app1.pdf]

App use		Gender identity, *n* (%)				Age (years), *n* (%)					
	Overall (*n*=761)	Females (*n*=668)	Males (*n*=84)	P value[a]	Effect size, Phi	18-25 (*n*=243)	>25-30 (*n*=115)	>30-40 (*n*=190)	>40 (*n*=208)	P value[a]	Effect size, Phi
**Name of app**											
Fitbit	171 (22.5)	158 (24.0)	9 (10.7)	.40	0.42	46 (19.0)	27 (23.5)	42 (22.1)	54 (26.0)	.10	0.75
Strava	130 (17.1)	102 (15.0)	27 (32.1)	__[b]	__	23 (9.5)	21 (18.3)	36 (18.9)	49 (23.6)	__	__
Garmin	102 (13.4)	91 (14.0)	11 (13.1)	__	__	21 (8.6)	16 (14.0)	38 (20.0)	26 (12.5)	__	__
Samsung Health	76 (10.0)	66 (10.0)	7 (8.3)	__	__	29 (12)	10 (8.7)	16 (8.4)	20 (10.0)	__	__
Apple Health	45 (5.9)	39 (6.0)	5 (6.0)	__	__	25 (10.3)	4 (3.5)	7 (2.7)	8 (4.0)	__	__
MapMyRun or MapMyWalk or MapMyFitness	21 (2.8)	21 (3.1)	0 (0.0)	__	__	3 (1.2)	7 (6.1)	6 (3.2)	5 (2.4)	__	__
Runkeeper	18 (2.4)	15 (2.2)	3 (3.6)	__	__	5 (2.1)	2 (1.7)	5 (2.6)	6 (2.9)	__	__
MyFitnessPal	18 (2.4)	16 (2.4)	2 (2.4)	__	__	9 (3.7)	3 (2.6)	4 (2.11)	2 (1.0)	__	__
GoogleFit	14 (1.8)	9 (1.3)	3 (3.6)	__	__	6 (2.5)	4 (3.5)	3 (1.6)	1 (0.5)	__	__
MyZone	7 (0.9)	6 (0.9)	0 (0.0)	__	__	4 (1.6)	0 (0.0)	0 (0.0)	3 (1.4)	__	__
**Type of app**											
Tracking	693 (91.3)	607 (90.9)	77 (91.7)	.99	0.007	205 (84.4)	105 (91.3)	177 (93.2)	201 (96.6)	.55	0.19
Guided workouts	47 (6.2)	42 (6.3)	5 (6.0)	__	__	27 (11.1)	9 (7.8)	7 (3.7)	4 (1.9)	__	__
Tracking and workouts	10 (1.3)	9 (1.3)	1 (1.2)	__	__	4 (1.6)	1 (0.9)	3 (1.6)	2 (1.0)	__	__
Other (booking classes or immersive games)	9 (1.2)	8 (1.2)	1 (1.2)	__	__	6 (2.5)	0 (0.0)	3 (1.6)	0 (0.0)	__	__
**Physical activity app is used for**											
All daily activity	91 (12.0)	81 (12.1)	8 (9.5)	.18	0.09	31 (12.7)	16 (14.0)	24 (12.6)	18 (9.0)	.09	0.16
Individual activities	574 (75.4)	499 (74.7)	70 (83.3)	__	__	175 (72.0)	83 (72.2)	141 (74.2)	173 (83.2)	__	__
Group-based activities	5 (0.7)	5 (0.8)	0 (0.0)	__	__	3 (1.3)	1 (0.9)	1 (0.5)	1 (0.5)	__	__
Gym-based activities	41 (5.4)	39 (6.0)	2 (2.4)	__	__	19 (8.0)	10 (8.7)	6 (3.2)	6 (3.0)	__	__
Combination of individual and group-based and gym-based activities	22 (3.0)	22 (3.4)	0 (0.0)	__	__	8 (3.3)	2 (1.7)	8 (4.2)	4 (2.0)	__	__

[a]Statistical significance is represented by P<.05.

[b]Not applicable.
